# Diagnostic Validity of Clinical Signs Associated with a Large Exophoria at Near

**DOI:** 10.1155/2013/549435

**Published:** 2013-06-17

**Authors:** Pilar Cacho-Martínez, Ángel García-Muñoz, María Teresa Ruiz-Cantero

**Affiliations:** ^1^Departamento de Óptica, Farmacología y Anatomía, Apartado 99, Universidad de Alicante, 03080 Alicante, Spain; ^2^CIBERESP, Área de Medicina Preventiva y Salud Pública, Apartado 99, Universidad de Alicante, 03080 Alicante, Spain

## Abstract

*Purpose*. To analyze the diagnostic validity of accommodative and binocular tests in a sample of patients with a large near exophoria with moderate to severe symptoms. *Methods*. Two groups of patients between 19 and 35 years were recruited from a university clinic: 33 subjects with large exophoria at near vision and moderate or high visual discomfort and 33 patients with normal heterophoria and low visual discomfort. Visual discomfort was defined using the Conlon survey. A refractive exam and an exhaustive evaluation of accommodation and vergence were assessed. Diagnostic validity by means of receiver operator characteristic (ROC) curves, sensitivity (S), specificity (Sp), and positive and negative likelihood ratios (LR+, LR−) were assessed. This analysis was also carried out considering multiple tests as serial testing strategy. *Results*. ROC analysis showed the best diagnostic accuracy for receded near point of convergence (NPC) recovery (area = 0.929) and binocular accommodative facility (BAF) (area = 0.886). Using the cut-offs obtained with ROC analysis, the best diagnostic validity was obtained for the combination of NPC recovery and BAF (S  =  0.77, Sp = 1, LR+ = value tending to infinity, LR− = 0.23) and the combination of NPC break and recovery with BAF (S  =  0.73, Sp = 1, LR+ = tending to infinity, LR− = 0.27). *Conclusions*. NPC and BAF tests were the tests with the best diagnostic accuracy for subjects with large near exophoria and moderate to severe symptoms.

## 1. Introduction

Convergence insufficiency (CI) is a sensory motor anomaly that is characterized by an inability to accurately converge or sustain convergence at near, which can cause substantial symptomatology during reading and near visual tasks [1, 2]. It is a common vision anomaly usually characterized as a binocular vision disorder with a low AC/A ratio in which the patient may have an orthophoria or exophoria at distance, with a moderate to high exophoria at near, greater than the distance phoria [3, 4], reporting as clinical characteristics, several symptoms and signs that can be present during the visual examination [1, 5–10].

In recent years, several randomized clinical trials [11–14] have studied the effectiveness of treatments for CI in children and adults, showing that office-based vision therapy with home reinforcement is the most effective treatment for CI. In fact, several reviews have shown there is sufficient evidence to support the use of vision therapy for CI [15–18]. 

According to epidemiology, numerous studies have suggested that this nonstrabismic binocular vision disorder is commonly found in clinical practice [19–30]. However, several authors have recently shown that the prevalence of CI is not really known because no population-based studies are available [31, 32]. Cacho-Martínez et al. [32] have revealed in a systematic review that there is a great variability in the reported prevalence of CI, ranging from 2.25 to 33%. The wide discrepancies in prevalence figures obtained are due to both sample population (neither randomized nor representative) and the lack of uniformity in diagnostic criteria. Similarly, Cooper and Jamal [31] have also shown in a recent literature review that prevalence of CI has a great variability with the average prevalence reported to be approximately 5%. They state that this variability can be attributed to differences in the definitions of CI, the sample studied (clinic samples versus general population), and differences in testing protocols. Other studies have also shown that patients with traumatic brain injury (TBI) have a greater incidence rate [33]. CI is evident in up to 48% of veterans exposed to blast injuries [34–36] and in about 40% of the civilian population with TBI, predominantly from motor vehicle accidents and falls [37–39]. 

Throughout the years, numerous investigators have used diverse definitions in the diagnosis of CI [31] existing different clinical criteria for diagnosing this condition [1, 5–10]. In fact, when studying this anomaly there is not a particular clinical sign which may assure that a patient has CI so that, in general, clinicians use a battery of symptoms and signs which allow them its diagnosis. 

Symptoms are varied, usually associated with tasks at near vision [4] including asthenopia, headaches, eyestrain, intermittent blurred vision, intermittent diplopia, impossibility to maintain clear vision for a reasonable period of time, difficulty in reading, movement of letters, sleeping when reading, decreasing the comprehension of reading with time, and loss of concentration [1, 17, 22, 40–42]. These symptoms may negatively impact an individual's quality of life and daily activities such as employment [38] and schoolwork [43]. The association of CI and symptoms has been investigated by the Convergence Insufficiency Treatment Trial Study Group (CITT Study Group) who developed the Convergence Insufficiency Symptom Survey (CISS) [6–8]. It is a questionnaire with 15 questions designed to quantify the severity of symptoms associated with CI. Initial [6–8] and later studies [44] have confirmed the validity and reliability of the CISS V-15 for evaluating symptoms in adults and children with CI. Similarly, Conlon et al. [45] developed a survey to measure visual discomfort in adults. The survey, which consists of 23 items, has been shown to be a valid instrument to measure visual anomalies reported by subjects with visual discomfort [45, 46]. Borsting et al. [47] have also revealed that both the Conlon et al. survey [45] and the CISS V-15 [7, 8] are reliable to investigate the long-term variability of visual discomfort. They encountered that visual discomfort symptom reporting using the Conlon survey is stable in the majority of college students over a 1-year period, reporting a good intraclass correlation coefficient (0.82).

Several authors [1, 4–10] refer to different clinical signs during visual examination: a moderate or high exophoria at near (greater than at distance vision), reduced positive fusional vergence (PFV) at near, reduced vergence facility at near with base-out prisms, a receded near point of convergence (NPC), a binocular accommodative facility (BAF) reduced with +2.00 D, diminished MEM retinoscopy or low fused crossed cylinders, diminished negative relative accommodation (NRA), exofixation disparity at near vision, intermittent suppression at near vision, and even a limited stereopsis. Recently, a systematic review [48] about the evidence of diagnostic criteria for general binocular dysfunctions has shown the use of different number of clinical signs [1, 5–10] ranging from one to five tests. Although no one of the authors validates the tests used by comparison against an established reference standard (gold standard) [49], all of them agree to consider the large exophoria at near for diagnosing CI, being both the PFV (85.7%) and the receded NPC (71.4%) the other clinical tests most frequently used [48]. 

In this sense, the CITT group developed a classification scheme for CI based on the following signs: exophoria at near vision greater than distance, ≥4 prismatic diopters (Δ), receded NPC, and reduced PFV range [28]. This classification system, as the authors declare in their study, is based on the signs most often associated with CI and many investigators have used it for prevalence, diagnosis and treatment purposes [1, 6–8, 11–14, 18, 22, 28, 29, 40, 41, 44]. 

In addition to the great variety of clinical signs for CI, scientific literature [48] also shows differences on cut-offs points for different tests. The large near exophoria varies between authors from 5Δ [1, 6–8], >6Δ [9] to 16Δ [10]. Similarly, some investigators consider receded NPC, values those results for break NPC which are ≥6 cm [1, 7, 8], ≥7.5 cm [6] and >10/17.5 cm for break and recovery NPC, respectively [9]. According to PFV, most authors [1, 6–8] consider reduced PFV at near when patient fails to reach Sheard's criteria [4] or fails to have minimum normative at near (≤15Δ) for break. Others [9] consider a reduced value of PFV ≤ 11/14/3Δ or PFV = 0Δ [5].

Consequently, disparity of both clinical signs and cut-offs may provide unequal diagnoses among authors. In any case, the greater difficulty of existing studies about diagnosis of CI are the lack of epidemiological criteria to justify the use of several tests as well as their cut-offs. They do not analyze diagnostic validity of clinical signs using likelihood ratios, sensitivity, specificity, or receiver operator characteristic (ROC) curves. The authors diagnose based on the criteria they consider patients should have without justifying why certain clinical signs must be taken into account and others must not. 

Considering that CI is a nonstrabismic binocular anomaly associated with a large near exophoria [4], the aim of this study is to identify the accommodative and binocular tests which present anomalous values in a sample of patients with a large near exophoria with moderate to severe symptoms and to analyze their diagnostic validity by means of ROC analysis, sensitivity, specificity, and likelihood ratios. 

## 2. Material and Methods

### 2.1. Patients

A prospective study was conducted at the Optometric Clinic of University of Alicante, Spain. For those patients who were coming consecutively for a routine visual examination with ages between 18 and 35, binocular status was obtained using the cover test method. The upper limit of 35 years was to avoid including subjects with prepresbyopia [50]. The study followed the tenets of the Declaration of Helsinki, and informed consent was obtained from all subjects after explanation of the nature of the study. 

One experienced author (PCM) served as examiner to assess the cover test method for distance (6 m) and near vision (40 cm). The subject's subjective refraction was placed in a trial frame. Once evaluated the cover-uncover test to rule out patients with tropias at distance or near vision; the alternate cover test (ACT) protocol was then performed to evaluate the heterophoria status [51–56]. For objective procedure of prism neutralized ACT, each subject was instructed to fixate on a single letter of 20/30 visual acuity. Using a prism bar the phoria value was midway between the low and high neutral findings using an ACT.

Following the ACT, other examiner measured visual discomfort with Conlon et al. survey [45–47]. As we wanted to analyze a sample of patients with a large near exophoria and visual symptomatology but initially they did not have the CI diagnosis, a more general questionnaire than CISS V-15 one was used. Conlon survey consists of 23 items related to near tasks, asking the patient questions about the feeling of their eyes when reading or the presence of several symptoms as headache, diplopia, losing the place when reading, movement of letters, difficulty reading the words on a page, and having glare. Each item has a 4 point scale: 0: event never occurs, 1: occasionally, a couple of times a year, 2: Often, every few weeks, and 3: almost always, yielding scores ranging from 0 to 69. Once the patient has answered all items, the survey defines the following groups: low discomfort group (scored from 0 to 24), moderate discomfort group (scored from 25 to 48), and high visual discomfort (scored from 49 to 69). 

Taking into account ACT results and Conlon et al. scores [45], consecutive patients were divided into two groups: patients with large exophoria at near and moderate or high visual discomfort (EXO-MHVD) and patients with normal heterophoria and low visual discomfort (NH-LVD). The inclusion criteria for both groups of subjects are explained in Table 1. Following the inclusion criteria, 33 subjects with large exophoria [4, 57, 58] and moderate to high visual discomfort at near were selected. Their ages were ranging between 19 and 33 years, with a mean age of 24.76 ± 4.05 years. The sample population of the normal heterophoria and low visual discomfort group enrolled 33 persons with ages between 19 and 34 years with a mean age of 24.91 ± 3.95 years. 

Each subject of both groups received an exhaustive evaluation of accommodation and vergence. A battery of accommodative and binocular tests which determine the accommodative and vergence status of a patient were carried out while the subjects wore their subjective refractive exam in place. The following tests were performed. Monocular accommodative amplitude (AA) with push-up method [59, 60]. Monocular and binocular accommodative facility (MAF, BAF) was conducted following the procedure of Zellers et al. [61] at 40 cm using ±2.00 D flip lenses and a target with suppression control, evaluating if patient had difficulty focusing with plus or minus lenses. MEM dynamic retinoscopy at 40 cm with the result of the subjective exam placed in a trial frame and using trial lenses [62]. Positive and negative relative accommodations (PRA, NRA) while patient was fixating the horizontal line of 20/30 letters at 40 cm [63]. Positive fusional vergence at 40 cm with Risley prism (with a smooth gradual increase in prism power) using an accommodative target of 20/30 visual acuity [64] (VA). Break and recovery near point of convergence (NPC) using an accommodative target of 20/30 VA [65] at 40 cm while the subject was encouraged to try to keep the target single. Distance was calculated from the midsagittal plane of the patient's head to the nearest half centimeter. Vergence facility at 40 cm using loose prisms of 12Δ-base-out and 3Δ-base-in at 40 cm while fixating an accommodative target of 20/30 VA [66]. Gradient AC/A ratio using cover test and −1.00 D lenses [4]. Due to the importance of controlling accommodation during AC/A testing (as the accommodative response cannot be known) the patient was asked to maintain clarity of the test. Fusion with worth test and stereopsis with graded circles of Randot SO-002 test [4]. 

### 2.2. Epidemiology and Statistics

With the results of accommodative and binocular tests of both groups the Mann-Whitney *U* test for two independent samples was performed to detect if significant statistical differences (*P* < 0.05) between both groups were observed. A comparison between right and left eye was previously done for monocular tests. This analysis showed no significant differences between both eyes (*P* > 0.05), so that right eye results were only used. 

For those tests with significant statistical differences (*P* < 0.05), the diagnostic validity of the test was assessed by means of standard analyses: ROC curves, sensitivity (S), specificity (Sp), and positive and negative likelihood ratios (LR+, LR−) [49, 67]. 

Considering that in this study the presence of the condition is the large exophoria at near and moderate to severe symptoms, S is the proportion of patients of EXO-MHVD group who have a positive test result and Sp is the proportion of people of NH-LVD group who have a negative test result. 

LR is a measure [67] that allows for information about the diagnostic test itself to be summarized. LR+ shows how much to increase the probability of the condition if the test is positive, while the negative likelihood ratio (LR−) shows how much to decrease it if the test is negative. General guidelines suggest that an LR > 1 indicates an increased probability that the condition is present, and an LR < 1 indicates a decreased probability that the condition is present. 

A receiver operator characteristic (ROC) curve [49] plots the true positive rate (S) versus the false positive rate (1 − Sp) over a range of cut-off values. It is considered that the best cut-off point is at or near the “shoulder” of the ROC curve because as the sensitivity is progressively increased there is little or no loss in specificity until very high levels of sensitivity are achieved. Thus, the overall accuracy of a test can be described as the area under the ROC curve, so that the larger the area, the better the test. If this area has a value of 1 it will indicate the perfection of the test, as both values of S and Sp would be 1. 

In order to analyze which tests had the better diagnostic accuracy, for those tests which had obtained significant statistical differences (*P* < 0.05), the area under the ROC curve and the coordinates of the curve (the cut-off points for each test) were examined. The choice of these cut-off points was made by means of a balance between S and Sp. These cut-offs are necessary to take into account the number of patients who pass or fail each test. 

Once considered the diagnostic validity of each test separately, the same was carried out considering multiple tests as serial testing strategy. This situation implies that all tests must be present. For that, the order used was from the greater to the less accurate test considering the area under ROC curve. First of all it was considered that the subject failed the most accurate test. Secondly the subject failed the two tests with better area. Next the three tests with the better area and so on until taking into account all tests were analyzed. Once the combinations of tests with the best results were chosen, diagnostic validity was also performed using the cut-off derived from the normative values of the scientific literature. 

All the statistical and epidemiologic analysis was performed using the statistical software SPSS 15.0 for Windows and the EPIDAT 3.1 program. 

## 3. Results

Table 2 shows mean value and standard deviation for each accommodative and binocular test for both group of patients. Tests with statistically significant differences (*P* < 0.05) have been highlighted. According to BAF results, it was noted that all patients for EXO-MHVD Group had difficulty in focusing with positive lenses. 

**Figure 1:**
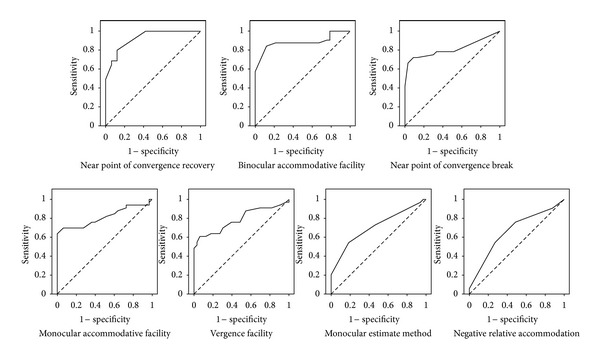
ROC curves for near point of convergence break and recovery, binocular accommodative facility, monocular accommodative facility, vergence facility, monocular estimate method and negative relative accommodation.

Figure 1 reveals ROC curves for each of the tests with statistical significant differences.  Table 3 shows the results of the area under the ROC curve for each clinical sign ordered from highest to lowest. The selected coordinates of each ROC curve, which represent the cut-off points for every test, appear in Table 4. Using these cut-off points, diagnostic validity was obtained for each test by means of S, Sp and LR ratios values with their confidence intervals to 95%; results are also shown in Table 4. 

Table 5 shows the results of S, Sp, LR+, and LR− considering multiple tests as serial testing strategy. As can be observed, the best results are obtained for the combination of both tests of NPC (break and recovery) and BAF which are those with the best diagnostic accuracy according to their ROC curves. Thus, once these three clinical signs were chosen and considering that the NPC has two responses, break and recovery point, three possible situations were considered. First, subjects failed the NPC break and BAF test having difficulty in focusing with positive lenses. Secondly, subjects failed NPC recovery with BAF. And thirdly, subjects failed NPC break and recovery and the BAF test.  Table 6 shows the diagnostic validity for these combinations using the cut-off points obtained by means of ROC curves and also considering the cut-off derived from the normative values of the scientific literature for NPC break and recovery [6, 28, 29, 68] and BAF testing [61].

## 4. Discussion

Results of this research have shown that the tests related to a near large exophoria having the better diagnostic accuracy are the NPC and BAF with difficulty in focusing with positive lenses. In any case, it is necessary to consider that these results may have limitations since the sample size is not too high. These findings could change in a higher sample of patients, in the sense that tests for which no statistical significant differences were detected (*P* > 0.05) could have been with a larger population.

Diagnostic validity considering cut-offs offered by ROC curves shows that the best results of S and Sp are for the NPC recovery with the cut-off of ≥8.25 cm. Similarly the test of BAF at the cut-off of ≤8.25 cycles per minute (cpm) achieves balanced values of S and Sp. Taking into account the peculiarity of NPC as the NPC recovery cannot be obtained without measuring previously the break value, it should be logical to also consider this result. The NPC break with the cut-off of ≥5.35 cm does not obtain a very high S but considering its balance with the Sp it is the value that allows a good Sp. 

Results of likelihood ratios show that NPC recovery, BAF, and NPC break are the tests with better diagnostic validity as they have a good balance between S, Sp, LR+, and LR−. Other tests as MAF obtain good results for positive likelihood. However, the negative likelihood is poor and the sensitivity is not very high. With these results, the selection of the NPC (break and recovery) and BAF as signs associated to the condition examined should be justified. Furthermore, these three tests have an area under the ROC curve close to 1. The fact that a test is more accurate when the area is larger would also justify the election of these clinical signs. 

When considering diagnostic validity of different combinations it can be observed that in all cases Sp reaches the value of 1, changing S and LR values. The best results are obtained for the combination of receded NPC recovery and BAF test failing with positive lenses. Reading these results implies that when both tests are used as serial testing strategy, that is, when the patient fails the NPC recovery, then the BAF is assessed and it fails having difficulty in focusing the image with positive lenses; 77% of subjects of EXO-MHVD group have a positive result. Furthermore, the SP achieved means that all subjects of NH-LVD group obtain adequate negative results as no one has a positive result in both tests. When considering likelihood ratios, LR+ result indicate that for EXO-MHVD group, there is a very high likelihood (a value which tends to infinity) of having a positive result (NPC and BAF failed) compared with the NH-LVD group. LR− of 0.23 indicates that for NH-LVD group, the likelihood of having a negative result (NPC and BAF normal) is 4.3 times greater than for EXO-NHVD group. When the NPC break is also considered (three clinical signs) results are also adequate. However, when assuming four clinical signs (adding MAF test), diagnostic validity results are poor. S and Sp values diminish and LR− of 0.42 indicates that for NH-LVD group, the likelihood of having a negative result is only 2.4 times greater than for EXO-NHVD group. This situation would justify the selection of NPC (recovery and break) and BAF testing not only for being the tests with the best area under the ROC curve but also because considering the combination of these three clinical signs adequate S, Sp, and LR ratios are obtained. In addition, results of this study also suggest that using the cut-off of ROC analysis, diagnostic validity is better than using the cut-off of scientific literature. 

According to the clinical signs associated with a large near exophoria, results of this study only partially coincide with the usual clinical signs associated with CI condition. [1, 5–14, 19–30, 69–78]. The finding of NPC as a clinical sign associated with the presence of a large near exophoria agrees with its use when diagnosing CI although the cut-off values differ between authors. The studies of Borsting et al. [6], Rouse et al. [28, 29], and Gallaway et al. [76] use a cut-off value of ≥7.5 cm for a receded NPC break. Several researches consider 6 cm to establish a receded NPC for CI [1, 7, 8, 11–14, 22, 30, 74]. However, others have used cut-offs of 10 cm [9, 20, 21, 24, 25, 69, 72, 73, 77] and some authors have considered 20 cm [26]. As it can be observed there are more studies which use the cut-offs of 6 cm and 10 cm even when only studies of adult population are considered [7, 9, 12, 24, 69, 73, 74, 76]. For NPC recovery, there are also differences between authors. Both studies of Rouse et al. [28, 29] use a cut-off value for NPC recovery ≥ 10.5 cm. Birnbaum et al. [69] use a value of >15 cm while those researches of Scheiman et al. [20] and García et al. [9] use the value of >17.5 cm. As it can be observed there are fewer authors who refer to NPC recovery for diagnosing CI. And even the authors who do use this clinical sign specify that the subject may fail the NPC break or recovery. 

It is clear that the cut-off value obtained in this study with ROC analysis for NPC break (≥5.35 cm) is lower than those used by other authors when CI is considered. However, it is more similar to those values found by other authors who have analyzed the NPC normative values. This is the case of the study of Scheiman et al. [65], in which the authors have found cut-offs of 5 cm for NPC break in an adult population with similar ages to those of this investigation, that is, a nonpresbyopic population. Similarly, Maples and Hoenes [79] recommend using an NPC break of ≥5 cm as a criterion to differ between asymptomatic and symptomatic subjects associated with the diagnosis of a CI. Nevertheless it is necessary to take into account that the authors [79] analyzed a sample of children with ages between 5 and 10 years and therefore not comparable with the adult population examined in our study. 

These comparisons cannot be established with other studies when considering the BAF test. Unlike what happens with the receded NPC, few studies explore BAF testing with difficulty focusing with positive lenses when analyzing CI, and when considering, authors mention it as a complementary sign which is not necessary to be present to diagnose the condition. This is the case of the studies of Lara et al. [24], Scheiman et al. [20], García et al. [9], and Shin et al. [30]. The difficulty on BAF testing with plus lenses should be related to low PFV finding, which has shown a frequent clinical sign associated with CI [48]. However this study does not show differences between both groups of adult patients so that the reduced PFV cannot be associated with a large near exophoria. This finding could be explained due to the small sample which may diminish the statistical power of results. A larger sample could have shown statistical differences between groups. Other explanation should be related to the fact that PFV measurements have shown low repeatability [80]. Anyway, the fact that BAF testing with difficulty in positive lenses has good diagnostic validity should indicate that subjects with a large exophoria at near may have altered the phasic component of the accommodative controller and not only exhibit a rapid adaptation of accommodation, as it has been stated by several authors [81]. 

In summary, this study shows that for subjects with a large near exophoria and moderate to severe symptoms, the accommodative and binocular tests that show a higher diagnostic accuracy are NPC and BAF. Then, when symptomatic adults present a large near exophoria and the clinician suspects a CI condition, it should be considered to measure the NPC. If the result is failed at break, recovery or both values the clinician should consider assessing the BAF testing with ±2.00 D. 

Although results of this study are based on a limited number of subjects and should be confirmed in forthcoming studies, they have important clinical implications. This is an investigation in which epidemiological tools have been used to identify which clinical signs are associated with a large near exophoria by means of diagnostic validity measurements. Accordingly, these findings may add evidence to support the importance of using different clinical tests in the assessment of binocular function in clinical settings. 

## Figures and Tables

**Table 1:** Inclusion criteria for EXO-MHVD and NH-LVD groups.

EXO-MHVD group	NH-LVD group
A score of 24 or higher on Conlon survey [45] was considered as moderate to severe symptoms	A score lower than 24 on Conlon survey [45] was considered as low symptoms
Near exophoria >6Δ. As the expected value of near phoria [4, 57, 58] is between a range of ortophoria and 6Δ of exophoria, this limit was selected to consider having a large value of near exophoria	Normative values for distance and near phoria [4, 57, 58]
Normative values of distance phoria [57, 58], or having a difference between both distance and near phoria out of a range of 5Δ [4]	Far and near visual acuity ≥20/20 with the best prescription, without ocular motility disorders, vertical deviation, strabismus or ocular pathology
Far and near visual acuity ≥20/20 with the best prescription, without ocular motility disorders, vertical deviation, strabismus or any type of ocular pathology	

**Table 2:** Comparison of samples between both groups of patients.

Test	NH-LVD group (N = 33) Average value ± SD	EXO-MHVD group (N = 33) Average value ± SD	*P*-value
AC/A	2.41/1 ± 1.31 Δ/D	1.96 ± 0.84 Δ/D	0.25
AA	10.97 ± 1.71 D	11.04 ± 2.03 D	0.83
MAF	12.86 ± 3.34 cpm	7.28 ± 5.29 cpm	<0.001*
BAF	10.82 ± 3.62 cpm	4.45 ± 4.14 cpm	<0.001*
MEM	0.61 ± 0.23 D	0.34 ± 0.37 D	0.002*
NRA	2.30 ± 0.32 D	2.07 ± 0.43 D	0.02*
PRA	3.56 ± 1.13 D	4.23 ±1.54 D	0.10
PFV blur	19.68 ± 6.11 Δ	16.70 ± 6.46 Δ	0.09
PFV break	25.64 ± 7.05 Δ	22.85 ± 8.42 Δ	0.10
PFV recovery	13.61 ± 6.35 Δ	13.73 ± 7.24 Δ	0.90
NPC break	2.93 ± 2.02 cm	7.00 ± 4.13 cm	<0.001*
NPC recovery	7.41 ± 1.19 cm	11.07 ± 3.05 cm	<0.001*
VF	15.91 ± 2.57 cpm	10.35 ± 6.16 cpm	<0.001*
Stereopsis	42.73 ± 9.45^#^	39.85 ± 9.23^#^	0.31

NH-LVD: normal heterophoria and low visual discomfort; EXO-MHVD: large exophoria at near and moderate or high visual discomfort; SD: standard deviation, AC/A: ratio AC/A, AA: accommodative amplitude, MAF: monocular accommodative facility, MEM: monocular estimated method, NRA: negative relative accommodation, PRA: positive relative accommodation, BAF: binocular accommodative facility, VF: vergence facility, PFV: positive fusional vergence, NPC: near point of convergence, Δ: prismatic diopter, D: diopter, cpm: cycles per minute, (^#^): seconds of arch. (*P < 0.05 indicates statistically significant differences between both groups).

**Table 3:** Area under the ROC curve for different tests.

Variable	Area	Confidence interval to 95 %		*P*-value
		Low limit	Top limit	
NPC recovery	0.929	0.855	1	<0.001
BAF	0.886	0.797	0.976	<0.001
NPC break	0.816	0.704	0.928	<0.001
MAF	0.814	0.704	0.925	<0.001
VF	0.787	0.672	0.901	<0.001
MEM	0.714	0.589	0.839	0.003
NRA	0.665	0.533	0.797	0.02

NPC: near point of convergence, BAF: binocular accommodative facility, MAF: monocular accommodative facility, VF: vergence facility, MEM: monocular estimate method, NRA: negative relative accommodation, *P* < 0.05: the obtained area differs statistically from the real value of 0.5.

**Table 4:** Diagnostic validity for each test using cut-offs obtained with ROC curves.

Test	Cut-off with ROC curve	Sensitivity (CI 95%)	Specificity (CI 95%)	LR+ (CI 95%)	LR− (CI 95%)
NPC recovery	≥8.25 cm	0.85 (0.69–1)	0.82 (0.61–1)	4.79 (1.69–14)	0.19 (0.08–0.48)
BAF	≤8.25 cpm	0.88 (0.75–1)	0.79 (0.63–0.94)	4.14 (2.12–8.09)	0.15 (0.06–0.39)
NPC break	≥5.35 cm	0.73 (0.56–0.89)	0.91 (0.80–1)	8.00 (2.67–24)	0.30 (0.17–0.53)
MAF	≤8.25 cpm	0.70 (0.52–0.87)	0.94 (0.84–1)	12.00 (2.95–45)	0.32 (0.19–0.55)
VF	≤14.75 cpm	0.70 (0.52–0.87)	0.70 (0.52–0.87)	2.30 (1.31–4.04)	0.25 (0.25–0.76)
MEM	≤0.63 D	0.73 (0.56–0.89)	0.55 (0.36–0.73)	1.60 (1.04–2.46)	0.50 (0.26–0.95)
NRA	≤2.38 D	0.76 (0.60–0.92)	0.52 (0.33–0.70)	2.30 (1.31–4.04)	0.43 (0.25–0.76)

LR+: positive likelihood ratio, LR−: negative likelihood ratio, CI: confidence interval, NPC: near point of convergence, BAF: binocular accommodative facility, MAF: monocular accommodative facility, VF: vergence facility, MEM: monocular estimated method, NRA: negative relative accommodation, cpm: cycles per minute, D: diopter.

**Table 5:** Diagnostic validity for different test combinations using cut-offs derived from ROC analysis.

Tests	Cut-off used	Sensitivity (CI 95%)	Specificity (CI 95%)	LR+ (CI 95%)	LR− (CI 95%)
NPC recovery	NPC recovery ≥ 8.25 cm	0.85 (0.69–1)	0.82 (0.61–1)	4.79 (1.69–14)	0.19 (0.08–0.48)
NPC recovery + BAF	NPC recovery ≥ 8.25 cm BAF ≤ 8.25 cpm	0.77 (0.59–0.95)	1 (0.97–1)	NA	0.23 (0.13–0.49)
NPC recovery + BAF + NPC break	NPC recovery ≥ 8.25 cm BAF ≤ 8.25 cpm NPC break ≥ 5.35 cm	0.73 (0.54–0.92)	1 (0.97–1)	NA	0.27 (0.15–0.53)
NPC recovery + BAF +NPC break + MAF	NPC recovery ≥ 8.25 cm BAF ≤ 8.25 cpm NPC break ≥ 5.35 cm MAF ≤ 8.25 cpm	0.58 (0.37–0.79)	1 (0.97–1)	NA	0.42 (0.28–0.68)
NPC recovery + BAF + NPC break + MAF + VF	NPC recovery ≥ 8.25 cm BAF ≤ 8.25 cpm NPC break ≥ 5.35 cm MAF ≤ 8.25 cpm VF ≤ 14.75 cpm	0.42 (0.21–0.63)	1 (0.97–1)	NA	0.58 (0.42–0.82)
NPC recovery + BAF +NPC break + MAF + VF + MEM	NPC recovery ≥ 8.25 cm BAF ≤ 8.25 cpm NPC break ≥ 5.35 cm MAF ≤ 8.25 cpm VF ≤ 14.75 cpm MEM ≤ 0.63 D	0.31 (0.11–0.50)	1 (0.97–1)	NA	0.69 (0.54–0.92)
NPC recovery + BAF +NPC break + MAF +VF + MEM + NRA	NPC recovery ≥ 8.25 cm BAF ≤ 8.25 cpm NPC break ≥ 5.35 cm MAF ≤ 8.25 cpm VF ≤ 14.75 cpm MEM ≤ 0.63 D NRA ≤ 2.38 D	0.27 (0.08–0.46)	1 (0.97–1)	NA	0.73 (0.58–0.95)

LR+: positive likelihood ratio, LR−: negative likelihood ratio, CI: confidence interval, NPC: near point of convergence, BAF: binocular accommodative facility, MAF: monocular accommodative facility, VF: vergence facility, MEM: monocular estimated method, NRA: negative relative accommodation, cpm: cycles per minute, D: diopter. NA: not applicable as the value tends to infinity.

**Table 6:** Diagnostic validity considering multiple tests as serial testing strategy using cut-offs derived from ROC analysis and scientific literature.

Tests	Cut-offs used	Sensitivity (CI 95%)	Specificity (CI 95%)	LR+ (CI 95%)	LR− (CI 95%)
NPC break + BAF	ROC NPC break ≥ 5.35 cm BAF ≤ 8.25 cpm	0.67 (0.49–0.84)	1 (0.98–1)	NA	0.33 (0.21–0.55)
NPC recovery + BAF	ROC NPC recovery ≥ 8.25 cm BAF ≤ 8.25 cpm	0.77 (0.59–0.95)	1 (0.97–1)	NA	0.23 (0.13–0.49)
NPC break + NPC recovery + BAF	ROC NPC break ≥ 5.35 cm NPC recovery ≥ 8.25 cm BAF ≤ 8.25 cpm	0.73 (0.54–0.92)	1 (0.97–1)	NA	0.27 (0.15–0.53)
NPC break + BAF	Literature NPC break ≥ 7.50 cm BAF < 3 cpm	0.21 (0.06–0.37)	1 (0.98–1)	NA	0.79 (0.66–0.95)
NPC recovery + BAF	Literature NPC recovery ≥ 10.50 cm BAF < 3 cpm	0.19 (0.02–0.36)	1 (0.97–1)	NA	0.81 (0.67–1)
NPC break + NPC recovery + BAF	Literature NPC break ≥ 7.50 cm NPC recovery ≥ 10.50 cm BAF < 3 cpm	0.19 (0.02–0.36)	1 (0.97–1)	NA	0.81 (0.67–1)

LR+: positive likelihood ratio, LR−: negative likelihood ratio, CI: confidence interval, NPC: near point of convergence, BAF: binocular accommodative facility, cpm: cycles per minute. NA: not applicable as the value tends to infinity.
